# The influence of psychological factors on coronary heart disease: A review of the evidence and implications for psychological interventions

**DOI:** 10.1097/MD.0000000000034248

**Published:** 2023-07-07

**Authors:** Weize Xu, Lingyan Fang, Hemanyun Bai, Kangling Ke, Weiyan Li, Haitao Huang, Xiao Liang, Can Chen

**Affiliations:** a Department of Cardiology, the Second Affiliated Hospital of Guangdong Medical University, Zhanjiang, Guangdong, China; b Guangdong Medical University, Zhanjiang, Guangdong, China.

**Keywords:** anxiety, coronary heart disease, depression, psychological factors, psychological interventions, social support, stress

## Abstract

This article reviews the evidence on the influence of psychological factors on coronary heart disease (CHD) and discusses the implications of these findings for psychological interventions. The review focuses on the role of work stress, depression, anxiety, and social support in the impact of CHD, as well as the effects of psychological interventions on CHD. The article concludes with recommendations for future research and clinical practice.

## 1. Introduction

Coronary heart disease (CHD) is a type of cardiovascular disease that occurs when plaque builds up inside the arteries that supply blood to the heart muscle. This plaque buildup, called atherosclerosis, can cause the arteries to narrow or become blocked, reducing blood flow to the heart. As a result, the heart may not get the oxygen and nutrients it needs to function properly, which can lead to chest pain or discomfort (angina), heart attack, or even death. CHD is a common and serious condition that affects millions of people worldwide and is a leading cause of death globally. Risk factors for CHD include high blood pressure, high cholesterol, smoking, diabetes, obesity, a family history of the disease, and a sedentary lifestyle. Management of CHD typically involves lifestyle changes (such as healthy eating and exercise), medications to manage risk factors, and, in some cases, medical procedures or surgery.^[1–3]^ Recent researches have highlighted the role of psychological factors in the development and progression of the disease. Psychological factors, such as depression, anxiety, stress, and personality traits, have been shown to have a significant impact on the risk of developing CHD and on outcomes following a diagnosis of the disease.^[4–6]^ Thus, there is a growing need for effective psychological interventions that can address the complex interplay between biological, psychological, and social factors that influence CHD. Such interventions can help individuals with CHD better cope with the disease, improve their quality of life, and reduce the risk of adverse health outcomes.

## 2. Methods

A systematic review of published literature was conducted using various electronic databases, including PubMed, Embase, and Web of Science, from inception to January 2023. The inclusion criteria were studies investigating the influence of psychological factors on CHD in human subjects with stress, depression, anxiety, and social support. The exclusion criteria were studies investigating the effects of other clinic interventions or studies not related to CHD. A thorough review of literature was conducted from a contemporary perspective, without concern for ethical issues.

### 2.1. Work stress on CHD

Stress at work refers to the physical, emotional, and mental strain experienced by employees in response to work-related demands or pressures. Numerous prospective cohort studies have investigated the association between work stress and CHD that is a result of an incident. The results of these studies on the effects of job stress have been described in the form of narrative reviews.^[7–9]^ And recently also in quantitative meta-analyses or a systemic review.^[10]^ A recent article provides a comprehensive and informative review of the existing literature on the relationship between work stress and CHD.^[11]^ About the article, authors examine various physiological and behavioral mechanisms that may contribute to the association between work stress and CHD, one of the strengths of this article is the in-depth discussion of the role of the hypothalamic-pituitary-adrenal axis in mediating the effects of work stress on physiological responses and CHD risk. The authors provide a detailed explanation of how chronic activation of the hypothalamic-pituitary-adrenal axis can lead to dysregulation of stress responses, resulting in increased cortisol levels, inflammation, and oxidative stress, which are all linked to CHD. Another strength of this article is the consideration of behavioral factors in the association between work stress and CHD.^[12]^ The above authors of the article highlight the role of unhealthy behaviors, such as smoking, physical inactivity, and poor diet, in the development of CHD. They also suggest that work-related stress can contribute to the adoption and maintenance of these unhealthy behaviors, which can further increase the risk of CHD, both individual and organizational interventions can be effective in reducing work-related stress. Individual interventions, such as stress management and relaxation techniques, may help reduce the negative physiological and behavioral responses to work stress. Organizational interventions, such as job redesign and work schedule flexibility, may also be effective in reducing work-related stress. The article also provides insights into potential interventions to reduce the impact of work stress on CHD risk, detailed analysis of the physiological and behavioral mechanisms underlying this relationship and its discussion of potential interventions to reduce CHD risk make it a valuable resource for researchers and healthcare professionals interested in this study.

### 2.2. Depression on CHD

Depression is a common mental health disorder characterized by persistent feelings of sadness, hopelessness, and loss of interest in activities. It can also lead to changes in appetite, sleep patterns, and physical symptoms like fatigue and aches. Depression can have a significant impact on an individual’s quality of life and daily functioning, affecting their ability to work, study, and maintain relationships. It is a leading cause of disability worldwide and can increase the risk of other physical health problems, such as cardiovascular disease.^[13,14]^ By Elísabet Alcocer-Gómez 1, Mario D Cordero et al (2017) explore the relationship between depression and cardiovascular diseases and specifically focuses on the role of the NLRP3 inflammasome in this relationship.^[14]^ Based on the article the authors first provide an overview of the prevalence and impact of both depression and cardiovascular disease, highlighting the need for effective treatment and prevention strategies, then delve into the role of the NLRP3 inflammasome, a protein complex involved in the immune response, in the development of both depression and cardiovascular disease. They also discuss the various mechanisms through which the NLRP3 inflammasome can contribute to these conditions, including activation of the immune system, oxidative stress, and endothelial dysfunction. In terms of immune system activation, the authors note that people with depression and cardiovascular disease often have higher levels of inflammatory markers, which can lead to chronic inflammation and damage to the cardiovascular system. The NLRP3 inflammasome is a key regulator of inflammation, and its activation has been linked to the development of both conditions, discussed the role of oxidative stress, which occurs when there is an imbalance between free radicals and antioxidants in the body. The NLRP3 inflammasome can contribute to oxidative stress by promoting the production of reactive oxygen species, which can damage cells and tissues. Finally, the authors discuss the potential therapeutic implications of targeting the NLRP3 inflammasome. They suggest that drugs that inhibit the inflammasome or reduce inflammation may be effective in treating both depression and cardiovascular disease. They also note that lifestyle interventions, such as exercise and stress reduction, may help to reduce inflammation and improve overall health. Overall, the article provides a comprehensive overview of the relationship between depression and cardiovascular disease, with a particular focus on the role of the NLRP3 inflammasome. The authors suggest that targeting the inflammasome may be a promising approach for preventing or treating both conditions and call for further research in this area. Other articles suggest that focuses specifically on the role of psychological interventions in managing CHD, particularly in relation to depression and authors review various types of interventions such as cognitive-behavioral therapy (CBT) and stress management, and discuss their effectiveness in reducing depressive symptoms and improving overall cardiovascular health,^[15,16]^ examines specifically the impact of depression on outcomes in patients with existing CHD and the authors review evidence suggesting that depression is associated with poorer outcomes such as increased mortality and higher rates of cardiovascular events.

### 2.3. Anxiety about CHD

Anxiety is a natural and common human emotion characterized by feelings of fear, nervousness, and unease about future uncertainties. While mild levels of anxiety can be normal and even helpful in certain situations, such as motivating a person to prepare for an important exam or presentation, excessive and prolonged anxiety can become problematic and interfere with daily life. Anxiety disorders are a group of mental health conditions characterized by persistent and excessive worry or fear that can interfere with daily activities, relationships, and overall quality of life. Examples of anxiety disorders include generalized anxiety disorder, panic disorder, social anxiety disorder, and specific phobias. Symptoms of anxiety can include physical sensations such as rapid heartbeat, sweating, trembling, and shortness of breath, as well as cognitive and emotional symptoms such as excessive worry, fear of losing control, and difficulty concentrating.^[17–20]^ Anxiety is associated with a variety of physical and psychological symptoms that can negatively impact health. Anxiety can lead to increased blood pressure, heart rate, and cortisol levels, which can contribute to the development and progression of CHD. A meta-analysis by Annelieke M Roest et al (2010) provides strong evidence for the association between anxiety and increased risk of incident CHD,^[21]^ the study suggests that individuals with anxiety are at higher risk of developing CHD compared to those without anxiety. Moreover, the study also shows that anxiety is a significant predictor of poor outcomes in patients with existing CHD, including higher rates of cardiovascular events and mortality. The authors of the study suggest that interventions targeting anxiety, such as CBT and exercise-based cardiac rehabilitation programs, may be effective in reducing the risk of CHD and improving outcomes in patients with existing CHD. And the study highlights the importance of considering psychological factors, such as anxiety, in the prevention and management of CHD. Similarly, a study conducted by Devereux et al (2012) provides evidence for the potential benefits of acute exercise in reducing anxiety ratings in patients with CHD and elevated anxiety.^[22]^ The findings of the article suggest that a single session of exercise can significantly reduce anxiety levels in these patients, providing a potential nonpharmacological approach to managing anxiety in this population, and the authors suggest that exercise-based interventions, such as cardiac rehabilitation programs, may be effective in reducing anxiety and improving cardiovascular outcomes in patients with CHD. However, further research is needed to fully understand the mechanisms underlying the link between anxiety and CHD and to develop more effective prevention and treatment strategies.

### 2.4. Social support on CHD

Social support, defined as the emotional, informational, or practical assistance provided by family, friends, and social networks, has been shown to be a significant predictor of health outcomes, including CHD.^[23–27]^ A review was based on the data of 2 cohorts of people aged 40 to 69 who were followed for about 15 years. The review evaluated the social support of people and other factors that affect lifestyle, and it used medical records to confirm the existence of stroke and CHD. It describes social support as “resources offered by others that one can use to cope with stressors.” This questionnaire examines different types of social support like emotional support, appraisal support, and instrumental support. The results of this review revealed that higher levels of social support were linked to a lower risk of CHD and stroke. It was also found that emotional support is especially efficient in decreasing the risk of both conditions and was revealed this connection to be consistent across all types of social support and across different subgroups within the population. The authors of the article discuss the potential ways in which the impact of social support is on health, such as by reducing stress, promoting healthy lifestyles, and enhancing immunity. They also acknowledge the drawbacks of the study including the lack of information about the changes in social support over time and the potential of unmeasured factors that may be confounding factors and recommend that interventions aimed towards enhancing social support could be an effective strategy for in reducing the likelihood of stroke and CHD, especially among those who are more at risk due to social isolation or other reasons. The review by Ai Ikeda et al (2008) was published.^[25]^

## 3. Psychological interventions on CHD

Psychological interventions refer to various therapeutic techniques or strategies used to address mental health, clinical disease, or emotional problems.^[28–31]^ These interventions are designed to help individuals develop coping skills, modify their thoughts and behaviors, manage stress, and improve their emotional well-being. Psychological interventions, including CBT, relaxation therapy, and stress management, can significantly reduce the risk of CHD and improve patient outcomes. These interventions are also found to be particularly effective in reducing depression, anxiety, and stress, all of which are risk factors for CHD. A meta-analysis of randomized controlled trials that assess the effectiveness of psychological interventions in improving outcomes for patients with CHD,^[32]^ and the study aimed to identify the impact of psychological interventions on clinical outcomes, health behaviors, and psychological outcomes in patients with CHD, and authors conducted a systematic review of 51 randomized controlled trials, which included a total of 9086 patients with CHD. And the interventions studied included CBT, relaxation therapy, stress management, and other psychological interventions. The outcomes by the interventions measured included mortality, hospitalizations, health behaviors (e.g., smoking cessation, physical activity, and diet), and psychological outcomes (e.g., depression, anxiety, and quality of life) which showed that psychological interventions had a small but statistically significant effect on reducing mortality and hospitalizations in patients with CHD. Additionally, psychological interventions were found to be effective in improving health behaviors, particularly in increasing physical activity and improving diet, and in reducing psychological distress such as depression and anxiety. The study also found that CBT had the greatest effect on reducing depression and anxiety, while relaxation therapy had the greatest effect on reducing blood pressure.

## 4. Conclusion

This article provides a comprehensive overview of the relationship between psychological factors and CHD. It highlights the important role that work stress, depression, anxiety, and social support play in the development and progression of CHD. Additionally, the article discusses the potential benefits of psychological interventions in reducing the risk of CHD and improving outcomes for those who already have the condition. The evidence presented in this review suggests that addressing psychological factors is a critical component of effective CHD prevention and management. Healthcare professionals should consider psychological factors as important risk factors for CHD and incorporate psychological interventions into treatment plans.

Further research is needed to better understand the mechanisms underlying the relationship between psychological factors and CHD and to identify the most effective psychological interventions for different patient populations. By continuing to investigate and address the role of psychological factors in CHD, we can improve the health and well-being of millions of people worldwide.

## Author contributions

**Conceptualization:** Weize Xu, Can Chen.

**Data curation:** Weize Xu, Lingyan Fang.

**Formal analysis:** Weize Xu, Lingyan Fang.

**Investigation:** Weize Xu, Hemanyun Bai, Kangling Ke.

**Methodology:** Weize Xu.

**Project administration:** Weize Xu, Lingyan Fang.

**Resources:** Weize Xu, Weiyan Li, Haitao Huang, Xiao Liang.

**Software:** Weize Xu.

**Supervision:** Weize Xu.

**Validation:** Weize Xu.

**Visualization:** Weize Xu, Lingyan Fang.

**Writing – original draft:** Weize Xu, Lingyan Fang.

**Writing – review & editing:** Weize Xu, Lingyan Fang.
